# REFEEDING SYNDROME IN A PATIENT WITH AN OBSTRUCTIVE PANCREATIC CANCER: AN EMERGING COMPLICATION OF ARTIFICIAL NUTRITION IN THE GASTROENTEROLOGY WARD

**DOI:** 10.1590/0102-672020210003e1589

**Published:** 2021-12-17

**Authors:** Marta PATITA, Gonçalo NUNES, Manuela CANHOTO, Jorge FONSECA

**Affiliations:** 1 Hospital Garcia de Orta, Gastroenterology, Almada, Almada, Portugal; 2 Universidade do Porto, ICBAS-UP, Instituto de Ciências Biomédicas Abel Salazar, Porto, Porto, Portugal; 3 CiiEM, Centro de Investigação Interdisciplinar Egas Moniz, PaMNEC, Grupo de Patologia Médica, Nutrição e Exercício Clínico, Almada, Monte da Caparica, Portugal

**Keywords:** Refeeding syndrome, Parenteral nutrition, Pancreatic neoplasms, Síndrome da realimentação, Nutrição parenteral, Neoplasias pancreáticas

## INTRODUCTION

Refeeding syndrome (RS) is a life-threatening condition first described in severe malnourished prisoners of the Second World War^1^. This syndrome is defined as electrolyte and fluid shifts associated with metabolic abnormalities developed during nutritional support. RS hallmark is hypophosphatemia, but also includes hypomagnesemia, hypokalemia, vitamin deficiencies, abnormal glucose metabolism and fluid retention. Prolonged fasting is the most important risk factor and RS may be precipitated by oral, enteral or parenteral nutrition^4^ ^,^ ^5^. 

The authors describe a case of RS in the gastroenterology ward exemplifying the importance of recognizing this underreported condition in patients with digestive pathology under nutritional therapy.

## CASE REPORT

**FIGURE 1:**
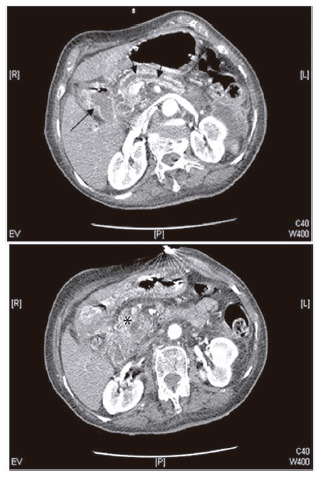
Abdominal computed tomography shows a heterogeneous mass of the head of the pancreas (*), which leads to marked dilatation of the Wirsung duct and duodenum (arrows)

An 82 year-old female was admitted due to recurrent vomiting during 10 days. Her past medical history included cerebrovascular disease, diabetes and hypertension. On hospital admission she was febrile, dehydrated and presented low body mass index (20.8 kg/m^2^). Initial evaluation revealed acute kidney injury (creatinine 4.1 mg/dl), hypokalaemia (K^+^ 3.2 mg/dl), hyperphosphatemia (Pi 5.4 mg/dl) and normal serum sodium and magnesium. Fluid and electrolyte replacement were immediately started but vomiting persisted. Nasogastric intubation revealed stasis (1800 cc/24h). Upper gastrointestinal endoscopy detected lumen narrowing in second/third duodenum parts. CT scan identified a heterogeneous mass in the pancreatic head causing Wirsung duct dilation and duodenal compression (Figure 1). Surgical resection was ruled out considering the advanced age and poor performance status. Palliative care with gastroduodenal self-expandable metallic stent (SEMS) placement was scheduled.

Since the patient displayed protein-energy malnutrition and the stent could not be placed immediately to resume oral feeding, total parenteral nutrition (TPN) was instituted through a central line, after correction of hypokalemia and preventive supplementation with intravenous phosphorus. Our gastroenterology department has a protocol for TPN gradual onset, starting with 25% of energy needs with progressive increase during the first week. However, TPN was started during the weekend, and the protocol was not applied due to an institutional mistake and reduced monitoring. Thus, 25 kcal/kg were administered during the first 24 h, which corresponded to 100% of energy needs. After the first day of TPN the patient developed depressed level of consciousness, myoclonus, sinus tachycardia, polypnea, pleural effusion and peripheral edema. Hypophosphatemia (Pi 0.6 mg/dl), worsening of hypokalemia (K^+^ 2.7 mg/dl), hypomagnesemia (Mg^2+^ 0.9mg/dl) and hypernatremia (Na^+^ 162 mg/dl) were detected in blood tests. RS diagnosis was assumed and the TPN prescription mistake was promptly identified. TPN was immediately stopped, thiamine supplementation and intensive intravenous hydration with electrolyte replacement and monitoring were implemented. Three days later, hydroelectrolytic balance was achieved and neurologic status recovered with complete resolution of clinical manifestations. Gastroduodenal SEMS was placed and oral feeding resumed. Refeeding was performed using an oral diet to supply initially 10% of the energy needs, reaching 100% at day 7, with no additional complications.

## DISCUSSION

Several disorders in the gastroenterology ward may induce significant weight-loss and fluid/electrolyte imbalance, including obstructive tumors causing dysphagia and vomiting. Nevertheless, RS remains poorly recognized with unknown incidence and no well-established diagnostic criteria^1^ ^,^ ^4^. Metabolic changes, in particular hypophosphatemia and hypokalaemia, may be life-threatening. Low serum phosphorus and potassium can induce severe cardiorespiratory events such as heart failure, arrhythmias and respiratory muscle weakness, and neurological abnormalities like paresthesia, myoclonus and seizures^4^.

To manage RS, most authors use the evidence described by National Institute for Health and Clinical Excellence (NICE) guidelines^2^. Actually, our patient presented high risk for RS given the low intake for at least 10 days and baseline low serum potassium^2,^ ^4^. Nutritional support should have been started, according with the protocol, with a maximum of 10 kcal/kg/day and increased slowly to achieve the total needs in 4-7 days. Close monitoring of electrolytes prior to begin TPN refeeding and during the first 10 days is of utmost importance. Although the impact of RS in patient outcome, hospitalization length and mortality are not established, some studies in critically ill showed increased mortality and longer admission when it develops and our experience with percutaneous endoscopic gastrostomy PEG-fed patients established increased mortality of hypophosphatemic patients^2^ ^,^ ^4^ ^,^ ^3^. This highlights the importance of RS awareness and the need of training for physicians who prescribe nutritional support and pharmacists who play an active role in selecting and preparing TPN bags^2^. The rules of avoiding initiating TPN by untrained staff and not to start during the weekend, when patients are not so closely monitored are highly advisable. The development of institutional protocols and multidisciplinary teams dedicated to nutritional support should be mandatory.
